# Stackelberg Dynamic Game-Based Resource Allocation in Threat Defense for Internet of Things

**DOI:** 10.3390/s18114074

**Published:** 2018-11-21

**Authors:** Bingjie Liu, Haitao Xu, Xianwei Zhou

**Affiliations:** School of Computer and Communication Engineering, University of Science and Technology Beijing, Beijing 100083, China; b20160290@xs.ustb.edu.cn (B.L.); xwzhouli@sina.com (X.Z.)

**Keywords:** resource allocation, threat defense, Internet of Things, Stackelberg dynamic game, Nash equilibrium

## Abstract

With the rapid development of the Internet of Things, there are a series of security problems faced by the IoT devices. As the IoT devices are generally devices with limited resources, how to effectively allocate the restricted resources facing the security problems is the key issue at present. In this paper, we study the resource allocation problem in threat defense for the resource-constrained IoT system, and propose a Stackelberg dynamic game model to get the optimal allocated resources for both the defender and attackers. The proposed Stackelberg dynamic game model is composed by one defender and many attackers. Given the objective functions of the defender and attackers, we analyze both the open-loop Nash equilibrium and feedback Nash equilibrium for the defender and attackers. Then both the defender and attackers can control their available resources based on the Nash equilibrium solutions of the dynamic game. Numerical simulation results show that correctness and effeteness of the proposed model.

## 1. Introduction

The Internet of Things (IoT) [1] refers to a huge network of various information-sensing devices combined with the Internet. These sensing devices include infrared sensors, Radio Frequency Identification Devices (RFID) [2], laser scanners, global positioning systems (GPS), and other devices. In recent years, with the development of computer intelligence technology, communication technology and perceptual recognition technology, the IoT has been widely used in smart home, smart medical, smart grid, Intelligent Transportation System (ITS) and other fields, and brought great convenience to people’s lives [3,4,5].

Generally, the IoT system is composed of a large number of nodes that are often exposed to public situations, lack effective protection, and are easily attacked [6,7,8]. Therefore, the security threats faced by IoT devices are more serious than those of the traditional network. In addition, the IoT environment is complex and IoT devices with limited resources are more vulnerable to cyber-attack [9]. Faced with limited resources, how to effectively allocate resources [10] to defend against threats in the IoT system has become a serious problem that desperately needs to be solved.

Lots of work has been done on resource allocation problems in threat defense for IoT systems. In order to build up the overall security of the IoT, studies [11,12] propose an overall security architecture for the IoT from different perspectives. In order to promote multiple resource-sharing and heterogeneous resource-demanding allocations, Intrusion Detection Systems (IDS) architecture and resource allocation are recommended [13]. Zhang et al. [14] evaluated the four levels of a security index system of the Internet of Things through fuzzy analytic hierarchy process, and concluded that the key indicators for improving the security of the Internet of Things are privacy protection, WSN anti-attack capability, intelligent node security, and information application security. Leusse et al. [15] analyzed the security threats of the IoT, and proposed a self-organized community security structure.

Since most IoT devices are micro-embedded devices, their hardware and software resources are very limited, and only a small number of computing tasks can be performed. There are not enough resources to implement defense against the attacks. For most of the time, the devices of the IoT system are unprotected and the resources of each device are limited. The authors of [16] developed a distributed algorithm to detect anomalous activities in the information flow in a wireless sensor network-based IoT system. Considering the limited computational and communication resources, Eschenauer et al. [17] proposed a key management scheme for the wireless sensor networks, which relies on probabilistic key sharing among the nodes of a random graph to make a trade-off between sensor memory cost and connectivity. The research into attack defense strategy plays a crucial role in defending against malicious attacks and protecting the security of the Internet of Things. Tague et al. [18] mapped the data traffic of the captured wireless mobile network and calculated the minimum cost of node capture attacks using a key protocol to enhance the security of data privacy. The literature [19,20,21] mainly focused on the research of node capture attacks on RFID and WSN in the IoT perception layer. Liu et al. [22] proposed a dynamic defense framework for IoT security. The literature [23] studies the optimal invasive differential game theory, which effectively reduces the intrusion frequency of intruders. Zhang et al. [24] proposed a lightweight defense algorithm for IoT network environment attacks.

Game theory is a particularly effective mathematical tool to study problems in diverse networks, such as power control in wireless networks [25,26], channel allocation in cognitive radio networks [27,28], congestion control in telecommunications networks [29], marketing and economics, and security problems [30,31]. In this paper, we propose Stackelberg dynamic game-based resource allocation model in threat defense for a resource-constrained IoT system. We will try to find optimal solutions for both the defender and attackers for resource allocation problems. In summary, the key contributions of this paper are as follows:

- Firstly, we research a cyber-security IoT system, which consists of one defender and N attackers. The defender tries to find the optimal allocated resources for threat defense, and the attackers try to use their resources to attack the IoT system.
- Secondly, a Stackelberg dynamic game model is proposed to formulate the resource allocation problem in threat defense for the Internet of Things. The Stackelberg game is a one-leader-many-followers game, where the defender is the leader and the attackers are the followers.
- For the dynamic game, we use the risk level as the system state. The objectives for the defender and attackers are to optimize the cost during the threat defense to find the optimal allocated resources for both the defender and attackers.
- Finally, the open-loop control solutions and the feedback control solutions for both the defender and attackers are given based on Bellman dynamic programming.

The remainder of the paper is organized as follows. Section 2 introduces the system model and problem formulation. Section 3 provides the Nash equilibrium solutions for the proposed game model. Numerical simulations are given in Section 4. Finally, we conclude the work in Section 5.

## 2. System Model and Problem Formulation 

We consider a cyber-security IoT system that is composed of one defender and N attackers. The defender can control its resources, such as energy resources, computing resources, and bandwidth resources, to resist intrusion from all sorts of attackers. The attackers will use all available resources to successfully break the defense and break into the IoT system. Based on these, we will try to formulate a dynamic model for both the defender and the attackers, to find their optimal strategies for resources allocation of the cyber-security IoT system in the process of defense and attack. In our proposed model, the relationship between the defender and the attackers is considered a one-leader-N-followers Stackelberg dynamic game, where the defender is the leader and the attackers are the followers. At the beginning of the Stackelberg dynamic game, the defender will choose a resource strategy to protect the system. After observing the defender’s strategy, the attackers will choose their optimal resource strategies for intrusion based on the observed defense strategy. Then the defender will allocate its resources to defense the invasion based on the attackers’ strategies. The strategies for both the defender and the attackers are dynamic and time-dependent.

In order to formulate the Stackelberg dynamic game, there should be a system state for both the defender and the attackers. As the risk level of the IoT system concerns both the defender and the attackers, we use it as the system state of the Stackelberg dynamic game. Generally speaking, the risk level is affected by the input variables, which are the defense strategy and the attack strategies. In our proposed model, we assume that the risk level is not only related to the current input variables, but also is affected by the current risk level with a degradation coefficient. Assuming $u_{0}\left(t\right)$ and $u_{i}\left(t\right)$ are the control variables of the defender and the attackers for the resource allocation, respectively, let x(t) denote the risk level of the IoT system, which can be given by the following differential equation [32]:

(1) $$\frac{dx\left(t\right)}{dt}=\alpha u_{0}\left(t\right)+\sum_{i\in N}\beta_{i}u_{i}\left(t\right)+\varepsilon x\left(t\right),$$

where α is a negative weighted factor and $\beta_{i}$ is a positive weighted factor that denote the strategies’ relative importance on the risk level. Through an effective defense strategy, the system will be more robust as time elapses. Then the risk level of the system will decrease with a random degradation coefficient, which is denoted by ε. Based on Equation (1), we find that, in our proposed IoT system, the risk level will decrease with the defender’s action, and increase with the attackers’ actions. Meanwhile, the longer the system continues, the lower the risk level, which is denoted by the random degradation coefficient ε.

Given the system state, we can now discuss the objective functions for the defender and the attackers. As the leader of the Stackelberg dynamic game, we will formulate the objective function for the defender first. The IoT system is usually composed of devices with limited resources, such as low power supply and limited battery capacity, so the defense cost is the main concern for the IoT system protection. For the defender, it aims to minimize the cost for protecting the IoT system and resisting the attackers with limited resources. Its objective function can be given as follows:

(2) $$U_{0}=\mu_{0}\sum_{i=1}^{N}u_{i}^{2}\left(t\right)+\nu_{0}u_{0}^{2}\left(t\right)+\rho_{0}\left(\tilde{x}-x\left(t\right)\right),$$

where $\mu_{0}$, $\nu_{0}$, and $\rho_{0}$ are positive weighted factors that denote the relative importance of the components. In our paper, we assume that the weighted factors add up to 1, which means the weighted factors are decimals larger than 0 and less than 1. The physical meanings of the weighted factors are the importance of the components in the cost function. The objective function of the defender has three components. The first part is $\sum_{i=1}^{N}u_{i}^{2}\left(t\right)$, which means the observing cost. The defender should observe the attackers’ strategies to generate its own strategy for system defense. The observing cost is a direct ratio to the attackers’ resource strategies; when the attackers spend more resources, the defender should pay more attention for observation. $u_{0}^{2}\left(t\right)$ is the second part of the objective function, and means the defending cost of the IoT system. Generally, the defending cost is directly related to the resources allocated for defending. The third part of the objective function of the defender is given by $\left(\tilde{x}-x\left(t\right)\right)$, which is the disparity between the maximum permissible risk level and the real-time risk level. The purpose of the defender is to reduce the risk level to ensure data security, even with a risk level of zero. Let $\tilde{x}$ denote the maximum permissible risk level; we should try to make the risk level no higher than the threshold. According to Equation (2), we find that the total instantaneous cost of the defender is a function of the allocated resources $u_{0}\left(t\right)$, $u_{i}\left(t\right)$, and the risk level x(t). The defender wants to find the optimal allocated resources $u_{0}\left(t\right)$ that minimize its cost function over time interval [0,T]:

(3) $$\underset{u_{0}\left(t\right)}{\min}L_{0}\left(t\right)=\underset{u_{0}\left(t\right)}{\min}\left\{\int_{0}^{T}\left\{\mu_{0}\sum_{i=1}^{N}u_{i}^{2}\left(t\right)+\nu_{0}u_{0}^{2}\left(t\right)+\rho_{0}\left(\tilde{x}-x\left(t\right)\right)\right\}e^{-rt}dt\right\},$$

subject to Equation (1). Here, r is the discount rate.

The attackers also want to invade the entire IoT system at a low cost. Therefore the aims of the attackers are to minimize the cost of breaking into the IoT system with limited resources. Its objective function can be given as follows:

(4) $$U_{i}=\mu_{i}u_{i}^{2}\left(t\right)+\nu_{i}u_{0}^{}\left(t\right)\left(u-u_{i}^{}\left(t\right)\right)+\rho_{i}\left(x\left(t\right)-\tilde{x}\right),$$

where $\mu_{i}$, $\nu_{i}$, and $\rho_{i}$ are positive weighted factors that denote the relative importance of the components. The objective function of the defender has three components. The first part is $u_{i}^{2}\left(t\right)$, the resource cost to the attacker. Generally, the attacker should allocate enough energy resources to attack the IoT system, and we use the linear quadratic form to denote the attacking resource cost, the energy or power cost during attack. The second part is the cost for observing and weakening the IoT system. We use $u-u_{i}^{}\left(t\right)$ to denote the resources available for observing, except for the allocated attacking resources, where u denotes the maximum resources that can be allocated. $u_{0}^{}\left(t\right)$ denotes the difficulty of weakening the IoT system; the more resources allocated for protecting by the defender, the harder it is to weaken the IoT system. We combine the above components to denote the system observing and weakening cost. The third part is the cost caused by the risk level, which is denoted by $\left(x\left(t\right)-\tilde{x}\right)$. Each attacker wants to increase the risk level of the IoT system higher than the maximum permissible risk level $\tilde{x}$. Based on the objective function, attacker i wants to find the optimal allocated resources $u_{i}^{}\left(t\right)$ that minimize its cost function over time interval [0,T]:

(5) $$\underset{u_{i}\left(t\right)}{\min}L_{i}\left(t\right)=\underset{u_{i}\left(t\right)}{\min}\left\{\int_{0}^{T}\left\{\mu_{i}u_{i}^{2}\left(t\right)+\nu_{i}u_{0}^{}\left(t\right)\left(u-u_{i}^{}\left(t\right)\right)+\rho_{i}\left(x\left(t\right)-\tilde{x}\right)\right\}e^{-rt}dt\right\},$$

subject to Equation (1). Here, r is the discount rate.

## 3. Game Analysis 

In this section, we will discuss both the open-loop Nash equilibrium solutions and the feedback Nash equilibrium solutions to the proposed game model established in the previous section, and analyze the optimal strategies for the attackers and defender in the IoT system. The open-loop Nash equilibrium solutions will be given first, followed by the feedback Nash equilibriums. Both solutions are given based on Bellman’s dynamic programming principle.

### 3.1. Open-Loop Nash Equilibrium Solutions

During the Stackelberg relations in the threat defense, the IoT system will first consume certain resources for implementing a defense strategy, then the attackers will attack the system based on the initial defending resource. After observing the attackers’ strategies, the resources of the IoT system will be recalibrated to cope with all kinds of risk. Because both the IoT system and attackers’ resources are limited, it is important to effectively allocate resources during the defense and attack based on the proposed Stackelberg dynamic game. If the defenders and attackers choose to commit their strategies from the outset, their information structure can be seen as an open-loop pattern, which means the optimal strategies for the defender and attackers are functions of the initial risk level x(0) and the time instant t. In this section, the open-loop Nash equilibrium will be given to the game Equations (3) and (5) to obtain the optimal strategies in a finite time horizon [0,T].

#### 3.1.1. Open-Loop Solutions for the Attackers

In order to minimize the cost function, each attacker needs to choose their optimal resource strategies based on the observed defense strategy. Before getting the optimal allocated resources, we first give some definitions for understanding the proposed game model.

**Definition** **1.** *For attacker*i*, the resource strategy*$u_{i}^{*}\left(t\right)$*is optimal if the following inequality holds for all feasible control*$u_{i}^{}\left(t\right)\neq u_{i}^{*}\left(t\right)$:

(6) $$L_{i}\left(u_{i}^{*}\left(t\right),x^{*}\left(t\right),t\right)\leq L_{i}\left(u_{i}\left(t\right),x\left(t\right),t\right).$$

**Definition** **2.** *A set of controls*$\left\{u_{i}^{*}\left(t\right)\right\}$*constitutes an open-loop Stackelberg equilibrium to the problem in Equation (5), and*$x^{*}\left(t\right)$*is the corresponding state trajectory, if there exists a costate function*$\Lambda_{i}^{}\left(t\right)$*such that the following relations are satisfied,*

(7) $$u_{i}^{*}\left(t\right)=\arg\underset{u_{i}\left(t\right)}{\min}\left\{U_{i}+\Lambda_{i}\left(t\right)\dot{x}\left(t\right)\right\},$$

(8) $${\dot{\Lambda }}_{i}\left(t\right)=-\frac{\partial \left[U_{i}+\Lambda_{i}\left(t\right)\dot{x}\left(t\right)\right]}{\partial x\left(t\right)},$$

*where*$\Lambda_{i}^{}\left(t\right)$*in Equation (8) is an adjoint equation to describe the dynamics of a costate variable. The costate function*$\Lambda_{i}^{}\left(t\right)$*is a function associated with the state variable*x(t)*. Generally, Equation (7) can be considered a Hamiltonian system*$H_{i}\left(t\right)$*of the proposed game model, and*$H_{i}\left(t\right)=U_{i}+\Lambda_{i}\left(t\right)\dot{x}\left(t\right)$.

Based on the definitions given above, we can solve the attacker’s optimal resource strategy problem based on the Bellman’s dynamic programming principle.

**Lemma** **1.** The optimal resource strategy to attacker i is

(9) $$u_{i}^{*}\left(t\right)=\frac{v_{i}u_{0}\left(t\right)-\beta_{i}\Lambda_{i}\left(t\right)}{2\mu_{i}},$$

where $\Lambda_{i}^{}\left(t\right)$ is given by the following:

(10) $$\Lambda_{i}\left(t\right)=\frac{e^{\varepsilon \left(t-T\right)}-\rho_{i}}{\varepsilon }.$$

**Proof.**  See Appendix A.

Equation (9) shows that the optimal resource strategy of the attacker will be affected by $u_{0}^{}\left(t\right)$ and costate functions $\Lambda_{i}^{}\left(t\right)$. We can see that the optimal resource strategy of attacker i is in positive proportion to the resource strategy $u_{0}^{}\left(t\right)$ of the defender. The attackers will choose their optimal resource strategies for intrusion based on the initial defense strategy.

#### 3.1.2. Open-Loop Solutions for the Defender

The defender will allocate its resources to defend the attackers based on the attackers’ strategies. In this subsection, we will give the open-loop Nash equilibrium to the defender.

**Definition** **3.** *For the defender, the resource strategy*$u_{0}^{*}\left(t\right)$*is optimal if the following inequality holds for all feasible control*$u_{0}^{}\left(t\right)\neq u_{0}^{*}\left(t\right)$:

(11) $$L_{0}\left(u_{0}^{*}\left(t\right),x^{*}\left(t\right),t\right)\leq L_{0}\left(u_{0}\left(t\right),x\left(t\right),t\right).$$

**Definition** **4.** *A set of controls* $\left\{u_{0}^{*}\left(t\right)\right\}$ *constitutes an open-loop Stackelberg equilibrium to the problem in Equation (3), and* $x^{*}\left(t\right)$ *is the corresponding state trajectory, if there exist costate functions* $\lambda_{0}^{}\left(t\right)$ *and* $\lambda_{i}^{}\left(t\right)$ *such that the following relations are satisfied:*

(12) $$u_{0}^{*}\left(t\right)=\arg\underset{u_{0}\left(t\right)}{\min}H_{0}\left(t\right),$$

(13) $${\dot{\lambda }}_{0}\left(t\right)=-\frac{\partial H_{0}\left(t\right)}{\partial x\left(t\right)},$$

(14) $${\dot{\lambda }}_{i}\left(t\right)=-\frac{\partial H_{0}\left(t\right)}{\partial \Lambda_{i}\left(t\right)},$$

*where the Hamiltonian system* $H_{0}\left(t\right)$ *of the defender can be expressed as follows:*

(15) $$\begin{array}{ll} H_{0}\left(t\right) & =U_{0}+\lambda_{0}\left(t\right)\dot{x}\left(t\right)+\sum_{i=1}^{N}\lambda_{i}\left(t\right){\dot{\Lambda }}_{i}\left(t\right)\\ & =\left[\mu_{0}\sum_{i=1}^{N}u_{i}^{2}\left(t\right)+\nu_{0}u_{0}^{2}\left(t\right)+\rho_{0}\left(\tilde{x}-x\left(t\right)\right)\right]\\ & +\lambda_{0}\left(t\right)\left[\alpha u_{0}\left(t\right)+\sum_{i\in N}\beta_{i}u_{i}\left(t\right)+\varepsilon x\left(t\right)\right]\\ & +\sum_{i=1}^{N}\lambda_{i}\left(t\right)\left(-\rho_{i}-\varepsilon \Lambda_{i}\left(t\right)\right) \end{array}.$$

Calculate the partial derivative for $\lambda_{0}^{}\left(t\right)$ and $\lambda_{i}^{}\left(t\right)$ in the Hamiltonian system $H_{0}\left(t\right)$, we can obtain,

(16) $${\dot{\lambda }}_{0}\left(t\right)=\rho_{0}-\varepsilon \lambda_{0}\left(t\right),$$

(17) $${\dot{\lambda }}_{i}\left(t\right)=\varepsilon \lambda_{i}\left(t\right).$$

Solving Equations (16) and (17), we have,

(18) $$\lambda_{0}\left(t\right)=\frac{\rho_{0}-e^{\varepsilon \left(t-T\right)}}{\varepsilon },$$

(19) $$\lambda_{i}\left(t\right)=\frac{e^{\varepsilon \left(T-t\right)}}{\varepsilon }.$$

Calculating the partial derivative for $u_{0}^{}\left(t\right)$ in Equation (15), we obtain,

(20) $$u_{0}^{*}\left(t\right)=-\frac{\alpha \lambda_{0}\left(t\right)}{2v_{0}}.$$

Based on the above analysis, the optimal solutions for both the attackers and defender are obtained, we get the corresponding state trajectory $x^{*}\left(t\right)$ using Equations (9) and (20) as follows:

(21) $$x_{}^{*}\left(t\right)=\frac{1}{\varepsilon }\left[e^{\varepsilon \left(T-t\right)}-\alpha u_{0}^{*}\left(t\right)-\sum_{i=1}^{N}\beta_{i}u_{i}^{*}\left(t\right)\;\right]=\frac{1}{\varepsilon }\left[e^{\varepsilon \left(T-t\right)}+\frac{\alpha^{2}\lambda_{0}\left(t\right)}{2v_{0}}-\sum_{i=1}^{N}\beta_{i}\frac{v_{i}u_{0}\left(t\right)-\beta_{i}\Lambda_{i}\left(t\right)}{2\mu_{i}}\right].$$

#### 3.1.3. Open-Loop Control Algorithm

In this subsection, we discuss the implementation open-loop control algorithm for the proposed game analysis. **Algorithm 1** is the open-loop control algorithm for the attackers and defenders. The whole algorithm cycling can be divided into two parts. One is the “open-loop control of attackers” part, which is used to calculate the optimal strategies of resource allocation during the attacks. The other is the “open-loop control of defender” part, to make a decision on the resource level for threat defense. The time complexity of the algorithm will be O(n), because the algorithm should be solved for all the attackers and the defender, and should be solved in a finite time horizon [0,T]. The space complexity of the presented solution is O(n), because the function for the open-loop solution should be invoked at each time. The progress can be described as follows.

**Algorithm 1.** Open-loop control algorithm for the attackers and defender.Start algorithm Step 1. Set up the parameter for the attackers and defender;Step 2. The defender controls its initial strategy for resource allocation for threat defense;Step 3. Start the open-loop control of the attackers and the defender;Step 4. Start to calculate the open-loop control solutions for the attackers,  Step 4.1. Set up the objective function for the attackers;  Step 4.2. Calculate the solutions for the attackers.Step 5. Get the open-loop solutions of the attackers for the defender;Step 6. Start to calculate the open-loop control solutions for the defender;  Step 6.1. Set up the objective function for the defender;  Step 6.2. Calculate the solutions for the defender.Algorithm End

### 3.2. Feedback Nash Equilibrium Solutions

To eliminate information nonuniqueness in the derivation of Nash equilibria, we can obtain the optimal solutions for the proposed game mode to satisfy the feedback Nash equilibrium property. In the feedback situation, the information structures of the defender and attackers follow a closed-loop perfect state pattern, and the optimal strategies for the defender and attackers become functions of the initial risk level x(t), the current risk level x(t) at time instant t, and the current time t. In this subsection, the feedback Nash equilibrium solutions to the proposed Stackelberg dynamic game are discussed based on the dynamic optimization programming technique developed by Bellman [33]. In the following subsections, we first discuss the optimal resource strategies for each attacker in a finite time horizon [0,T]. Then, the optimal strategy of the defender is obtained based on the attackers’ solutions.

#### 3.2.1. Feedback Solutions for the Attackers

In this section, we first discuss the optimal resource strategies for the attackers, the feedback Nash equilibrium solutions to the game Equations (1) and (5) will be discussed.

**Definition** **5.** *A set of control* $\left\{u_{i}^{*}\left(t\right)\right\}$ *constitutes a feedback solution to Equations (1) and (5); if there exists a continuously differentiable function* $V^{i}\left(t,x\right)$ *, and* $V^{i}\left(t,x\right)$ *satisfies the following differential equation:*

(22) $$-V_{t}^{i}\left(t,x\right)=\underset{u_{i}\left(t\right)}{\min}\left\{\left[\mu_{i}u_{i}^{2}\left(t\right)+\nu_{i}u_{0}^{}\left(t\right)\left(u-u_{i}^{}\left(t\right)\right)+\rho_{i}\left(x\left(t\right)-\tilde{x}\right)\right]e^{-rt}+V_{x}^{i}\left[\alpha u_{0}\left(t\right)+\sum_{i\in N}\beta_{i}u_{i}\left(t\right)+\varepsilon x\left(t\right)\right]\right\}.$$

Calculating the partial derivative for $u_{i}^{}\left(t\right)$ in Equation (22), we can then obtain

(23) $$u_{i}^{*}\left(t\right)=\frac{v_{i}u_{0}\left(t\right)-\beta_{i}V_{x}\left(t,x\right)e^{rt}}{2\mu_{i}}.$$

**Lemma** **2.** *The value function* $V^{i}\left(t,x\right)$ *admits a solution that satisfies,*

(24) $$V^{i}\left(t,x\right)=\left[A_{i}\left(t\right)x+B_{i}\left(t\right)\right]e^{-rt},$$

*where* $A_{i}\left(t\right)$ *is given by*

(25) $$A_{i}\left(t\right)=\frac{e^{\left(r-\varepsilon \right)\left(T-t\right)}+\rho_{i}}{r-\varepsilon },$$

*and* $B_{i}^{}\left(t\right)$ *are satisfied,*

(26) $$\begin{array}{l} {\dot{B}}_{i}\left(t\right)=rB_{i}\left(t\right)-A_{i}\left(t\right)\left[\alpha u_{0}\left(t\right)+\sum_{i\in N}\beta_{i}u_{i}\left(t\right)\right]\\ -\mu_{i}u_{i}^{2}\left(t\right)-\nu_{i}u_{0}^{}\left(t\right)\left(u-u_{i}^{}\left(t\right)\right)+\rho_{i}\tilde{x} \end{array}$$

**Proof.**  See Appendix B.

#### 3.2.2. Feedback Solutions for the Defender

In this subsection, the feedback Nash equilibrium solution for the defender will be discussed.

**Definition** **6.** *A set of control* $\left\{u_{0}^{*}\left(t\right)\right\}$ *constitutes an feedback solution to Equations (1) and (3), if there exists a continuously differentiable function* $V^{0}\left(t,x\right)$ *, and* $V^{0}\left(t,x\right)$ *satisfies the following differential equation:*

(27) $$-V_{t}^{0}\left(t,x\right)=\underset{u_{0}\left(t\right)}{\min}\left\{\left[\mu_{0}\sum_{i=1}^{N}u_{i}^{2}\left(t\right)+\nu_{0}u_{0}^{2}\left(t\right)+\rho_{0}\left(\tilde{x}-x\left(t\right)\right)\right]e^{-rt}+V_{x}^{0}\left[\alpha u_{0}\left(t\right)+\sum_{i\in N}\beta_{i}u_{i}\left(t\right)+\varepsilon x\left(t\right)\right]\right\}.$$

As the game leader, the defender should consider the resource strategies of the attackers before making a decision on the resource strategies. Calculating the partial derivative for $u_{0}^{}\left(t\right)$ in (27), we obtain

(28) $$u_{0}^{*}\left(t\right)=-\frac{\alpha V_{x}^{0}\left(t\right)e^{rt}}{2v_{0}}.$$

**Lemma** **3.** *The value function* $V^{0}\left(t,x\right)$ *admits a solution that satisfies,*

(29) $$V^{0}\left(t,x\right)=\left[A_{0}\left(t\right)x+B_{0}\left(t\right)\right]e^{-rt},$$

*where* $A_{0}\left(t\right)$ *and* $B_{0}\left(t\right)$ *are given by*

(30) $$\left\{ \begin{array}{l} {\dot{A}}_{0}\left(t\right)=\left(r-\varepsilon \right)A_{0}\left(t\right)+\rho_{0}\\ {\dot{B}}_{0}\left(t\right)=rB_{0}\left(t\right)-A_{0}\left(t\right)\left[\alpha u_{0}\left(t\right)+\sum_{i\in N}\beta_{i}u_{i}\left(t\right)\right]\\ -\mu_{0}\sum_{i=1}^{N}u_{i}^{2}\left(t\right)-\nu_{0}u_{0}^{2}\left(t\right)-\rho_{0}\tilde{x} \end{array}\right..$$

**Proof.**  By taking the derivative of $V^{0}\left(t,x\right)$ with respect to t and x, we obtain,

(31) $$V_{t}^{0}\left(t,x\right)=\left[-rA_{0}\left(t\right)+{\dot{A}}_{0}\left(t\right)\right]x+\left[-rB_{0}\left(t\right)+{\dot{B}}_{0}\left(t\right)\right],$$

(32) $$V_{x}^{0}\left(t,x\right)=A_{0}\left(t\right)e^{-rt}.$$

Solving Equations (27), (31), and (32), $A_{0}^{}\left(t\right)$ and $B_{0}^{}\left(t\right)$ are satisfied:

(33) $$\left\{ \begin{array}{l} {\dot{A}}_{0}\left(t\right)=\left(r-\varepsilon \right)A_{0}\left(t\right)+\rho_{0}\\ {\dot{B}}_{0}\left(t\right)=rB_{0}\left(t\right)-A_{0}\left(t\right)\left[\alpha u_{0}\left(t\right)+\sum_{i\in N}\beta_{i}u_{i}\left(t\right)\right]-\mu_{0}\sum_{i=1}^{N}u_{i}^{2}\left(t\right)-\nu_{0}u_{0}^{2}\left(t\right)-\rho_{0}\tilde{x} \end{array}\right..$$

Solving the above equation, we can obtain the expression of $A_{0}^{}\left(t\right)$ as follows:

(34) $$A_{0}\left(t\right)=\frac{e^{\left(r-\varepsilon \right)\left(T-t\right)}-\rho_{0}}{r-\varepsilon }.$$

Substituting Equation (34) into Equation (28), we can derive the optimal resource strategy for the defender as follows:

(35) $$u_{0}^{*}\left(t\right)=-\frac{\alpha A_{0}\left(t\right)}{2v_{0}}.$$

Solving Equation (1), we can get the optimal state:

(36) $$x_{}^{*}\left(t\right)=\frac{1}{\varepsilon }\left[e^{\varepsilon \left(T-t\right)}-\alpha u_{0}^{*}\left(t\right)-\sum_{i=1}^{N}\beta_{i}u_{i}^{*}\left(t\right)\;\right]=\frac{1}{\varepsilon }\left[e^{\varepsilon \left(T-t\right)}+\frac{\alpha^{2}A_{0}\left(t\right)}{2v_{0}}-\sum_{i=1}^{N}\beta_{i}\frac{v_{i}u_{0}\left(t\right)-\beta_{i}A_{i}\left(t\right)}{2\mu_{i}}\right].$$

#### 3.2.3. Feedback Control Algorithm

In this subsection, we will discuss the implementation feedback control algorithm for the proposed game analysis, which is given in **Algorithm 2**. Similarly, the whole algorithm cycling can be divided into the attackers’ part, and the defender’s part. The time complexity of the feedback control algorithm will be O(n), and the space complexity is O(n). The progress can be described as follows.

**Algorithm 2.** Feedback control algorithm for the attackers and defender.Start algorithm Step 1. Set up the parameter for the attackers and defender;Step 2. The defender control its initial strategy for resource allocation for threat defense;Step 3. Start the feedback control of the attackers and the defender;Step 4. Start to calculate the feedback control solutions for the attackers,  Step 4.1. Set up the objective function for the attackers;  Step 4.2. Calculate the solutions for the attackers.Step 5. Get the feedback solutions of the attackers for the defender;Step 6. Start to calculate the feedback control solutions for the defender;  Step 6.1. Set up the objective function for the defender;  Step 6.2. Calculate the solutions for the defender.Algorithm End

## 4. Numerical Simulations

In this section, we will use MATLAB software to simulate the proposed Stackelberg dynamic game model. We will analyze the resource strategies of attackers and defender, in the form of open-loop and feedback. The simulation parameters are shown in Table 1. To simplify the simulations, we assume all the attackers are uniform with the same simulation parameters.

### 4.1. Numerical Simulations of Open-Loop Nash Equilibrium Solutions

We first simulate the open-loop Nash equilibrium solutions of the model to get the optimal defense resource strategies of the defender and attackers.

**Figure 1:**
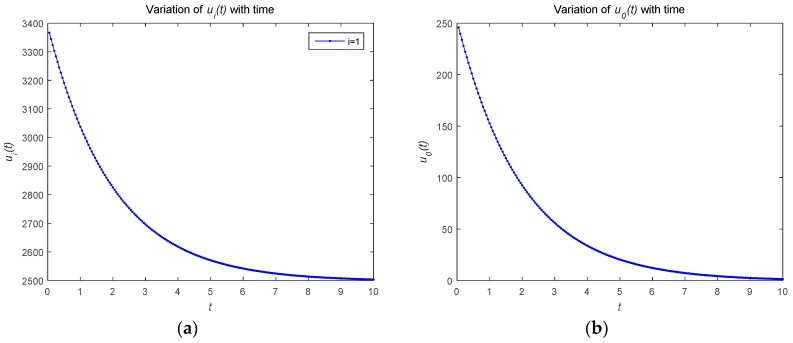
(**a**) Optimal strategy of the attackers; (**b**) optimal strategy of the defender.

Figure 1 describes the relationship between the optimal strategies $u_{i}^{*}\left(t\right)$ and $u_{0}^{*}\left(t\right)$ over time t(t∈[0,10]). As shown in Figure 1, the optimal resource strategy of both the attacker and defenders monotonically decrease with time t. In order to protect the security of the system, the defender adopts a strategy to consume its own resources when the attacker attacks. The attacker adopts a strategy to attack the system and consumes its own resources. As the time changes, the optimal strategies for the defender and attackers tend to convergence.

**Figure 2:**
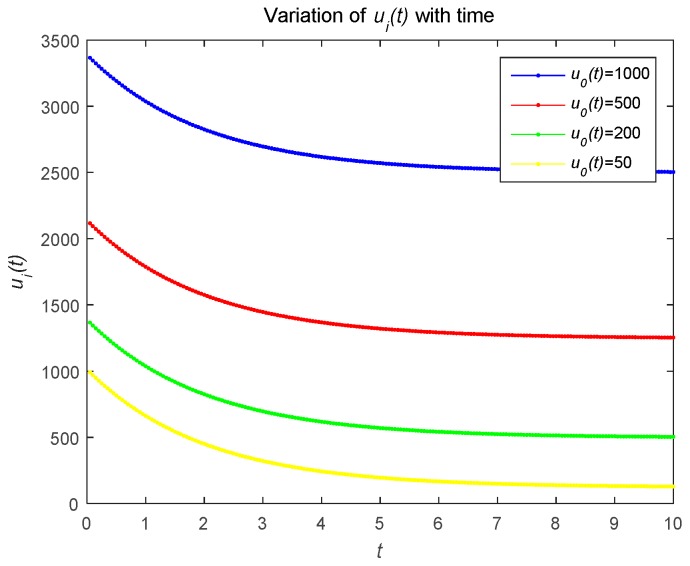
Optimal strategy of the attacker with different $u_{0}^{}\left(t\right)$ over time.

**Figure 3:**
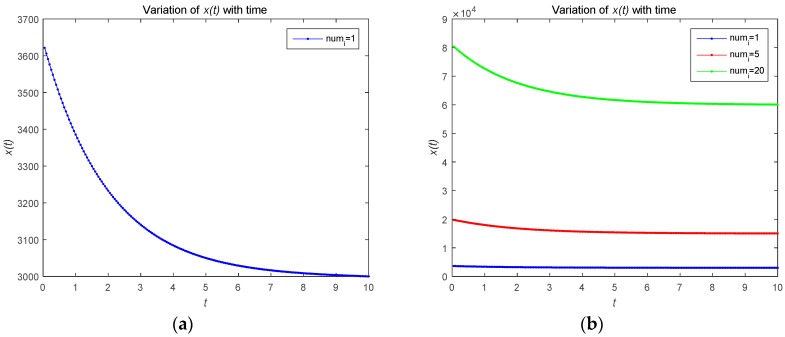
(**a**) Risk level variation for a system with one attacker; (**b**) risk level variation for a system with different numbers of attackers.

**Figure 4:**
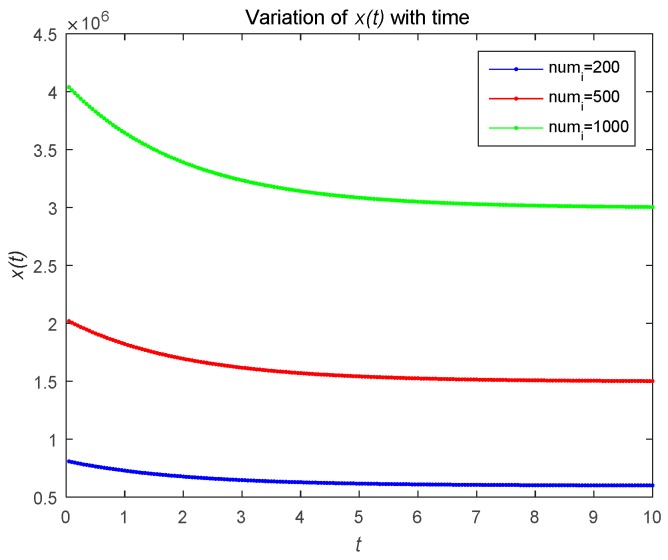
Risk level with a large number of attackers under open-loop control.

Figure 2 describes the changes in the attacker’s optimal resource strategy when $u_{0}^{}\left(t\right)$ takes different values. We find that the smaller $u_{0}^{}\left(t\right)$, the smaller the optimal resource strategy $u_{i}^{}\left(t\right)$. This is because the attacker will choose their optimal resource strategies for attacks based on the observed defense strategy. Figure 3 describes the relationship between the risk level of the system and time t. The risk level at the initial moment is the highest, and with the effective defense of the defender, the risk level shows a decreasing trend. As shown in Figure 3b, the risk level is a decreasing function with respect to time t, which is proportional to the number of attackers. The number of attackers are set to 1, 5, and 20, respectively. Figure 4 shows the risk level variation of the system, when the number of the devices in the IoT system becomes a large number, to analyze the scalability of the proposed model. We can prove that the proposed model can be used for IoTs with a large number of devices based on Figure 4.

### 4.2. Numerical Simulations of Feedback Nash Equilibrium Solutions

**Figure 5:**
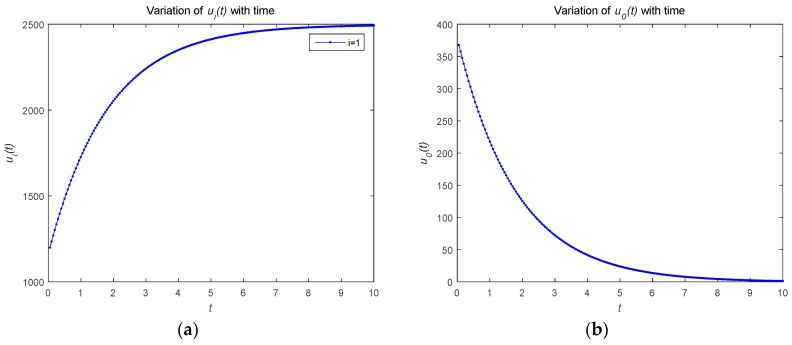
(**a**) Optimal strategy of the attacker; (**b**) optimal strategy of the defender.

This subsection mainly simulates the feedback Nash equilibrium solution of the model. Figure 5 describes the relationship between the optimal strategy $u_{i}^{*}\left(t\right)$ and $u_{0}^{*}\left(t\right)$ with time t(t∈[0,10]). As shown in Figure 5a, the attacker’s optimal resource strategy is an increasing function with respect to time t. The attackers control their own resource strategies. The aim of the attack is to increase the risk level, so they allocate more resources for attacks under the feedback control situation. As shown in Figure 5b, the defender’s optimal resource strategy is a decreasing function with respect to time t. The defender controls its own resource strategy to minimize risk, but, because of limited resources, may not have enough for defense as the time changes.

**Figure 6:**
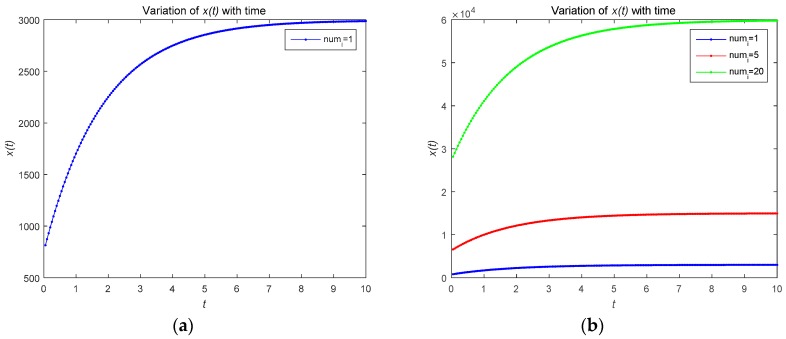
(**a**) Risk level variation for a system with one attacker; (**b**) risk level variation for a system with different numbers of attackers.

**Figure 7:**
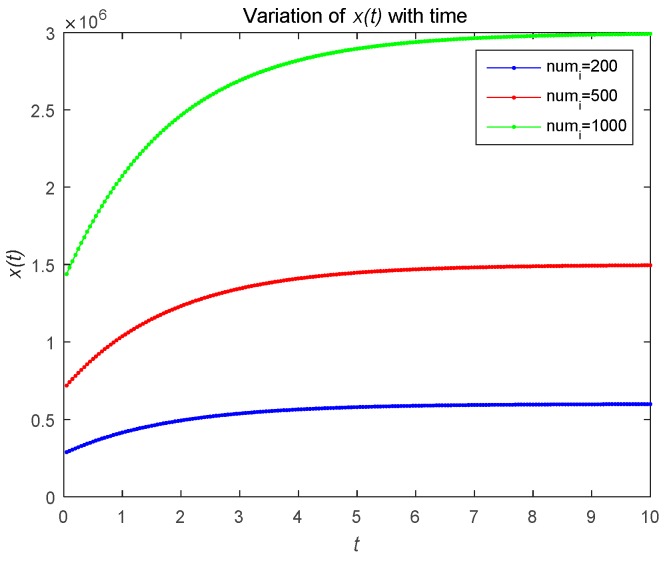
Risk level with a large number of attackers under feedback control.

**Figure 8:**
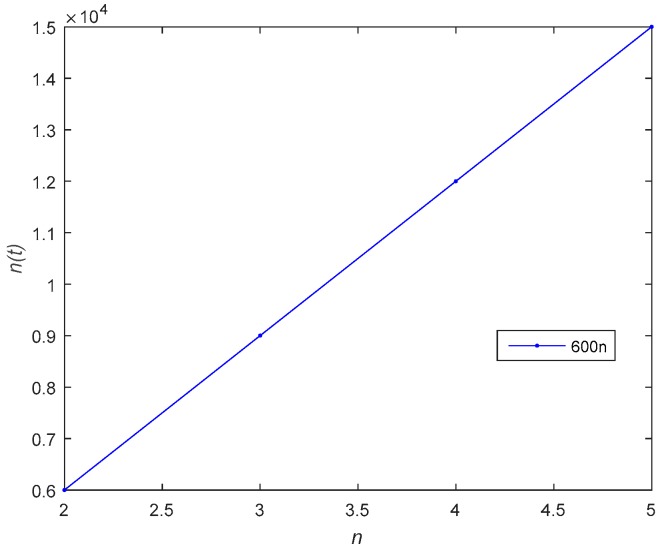
The time complexity of the proposed game model.

Figure 6 describes the relationship between the risk level of the system and time t. As shown in Figure 6, the risk level is proportional to the number of attackers. In the feedback Nash equilibrium solution, the attacker uses more attacks to increase the risk. Figure 7 shows the risk level variation of the system, when the number of devices in the IoT system becomes large. Figure 8 gives the time complexity of the proposed Stackelberg dynamic game. As shown in Figure 6, the time complexity of both the open-loop and feedback control algorithm will be O(n).

## 5. Conclusions

This paper proposes a Stackelberg dynamic game-based resource allocation model in the cyber-security IoT system that is composed by one defender and N attackers. We formulate a dynamic model for both the defender and the attackers to find their optimal strategies for resource allocation in the process of defense and attack. By solving the open-loop Nash equilibrium solution and the feedback Nash equilibrium solution, we find that the optimal resource solution for the defender is the open-loop Nash equilibrium solution, and under the open-loop situation, the defender can effectively reduce the risk level of the system. However, attackers can obtain more profit under the feedback situation.

## Figures and Tables

**Table 1:** Parameter settings.

Parameter	α	$\beta_{i}$	ε	$\mu_{0}$	$v_{0}$	$\rho_{0}$	$\mu_{i}$	$v_{i}$	$\rho_{i}$
Value	−0.85	0.6	−0.5	0.3	0.5	0.2	0.1	0.5	0.4

## Appendix A.

### ***Proof of Lemma 1.***

To solve the game model, we use the maximum principle proposed by Pontryagin et al. [34]. The Hamiltonian system of attacker i is given by

(37) $$\begin{array}{ll} H_{i}\left(t\right) & =U_{i}+\Lambda_{i}\left(t\right)\dot{x}\left(t\right)\\ & =\left[\mu_{i}u_{i}^{2}\left(t\right)+\nu_{i}u_{0}^{}\left(t\right)\left(u-u_{i}^{}\left(t\right)\right)+\rho_{i}\left(x\left(t\right)-\tilde{x}\right)\right]\\ & +\Lambda_{i}\left(t\right)\left[\alpha u_{0}\left(t\right)+\sum_{i\in N}\beta_{i}u_{i}\left(t\right)+\varepsilon x\left(t\right)\right] \end{array}$$

Calculating the partial derivative for $u_{i}^{}\left(t\right)$ and x(t) in Equation (37), we have

(38) $$\frac{\partial H_{i}\left(t\right)}{\partial u_{i}\left(t\right)}=2\mu_{i}u_{i}\left(t\right)-v_{i}u_{0}\left(t\right)+\beta_{i}\Lambda_{i}\left(t\right),$$

(39) $$\frac{\partial H_{i}\left(t\right)}{\partial x\left(t\right)}=\rho_{i}+\varepsilon \Lambda_{i}\left(t\right).$$

Then we have the optimal solution for resource allocation as follows:

(40) $$u_{i}^{*}\left(t\right)=\frac{v_{i}u_{0}\left(t\right)-\beta_{i}\Lambda_{i}\left(t\right)}{2\mu_{i}},$$

and the costate function $\Lambda_{i}^{}\left(t\right)$ can be given by the following differential equation,

(41) $${\dot{\Lambda }}_{i}\left(t\right)=-\rho_{i}-\varepsilon \Lambda_{i}\left(t\right).$$

Solving Equation (41), we can obtain the expression of the costate function $\Lambda_{i}^{}\left(t\right)$:

(42) $$\Lambda_{i}\left(t\right)=\frac{e^{\varepsilon \left(t-T\right)}-\rho_{i}}{\varepsilon }.$$

Hence, Lemma 1 follows.

## Appendix B.

### ***Proof of Lemma 2.***

By taking the derivative of $V^{i}\left(t,x\right)$ with respect to t and x, we obtain,

(43) $$V_{t}^{i}\left(t,x\right)=\left[-rA_{i}\left(t\right)+{\dot{A}}_{i}\left(t\right)\right]x+\left[-rB_{i}\left(t\right)+{\dot{B}}_{i}\left(t\right)\right],$$

(44) $$V_{x}^{i}\left(t,x\right)=A_{i}\left(t\right)e^{-rt}.$$

Substituting Equations (43–44) into Equation (27), $A_{i}^{}\left(t\right)$ and $B_{i}^{}\left(t\right)$ are satisfied:

(45) $$\left\{ \begin{array}{l} {\dot{A}}_{i}\left(t\right)=\left(r-\varepsilon \right)A_{i}\left(t\right)-\rho_{i}\\ {\dot{B}}_{i}\left(t\right)=rB_{i}\left(t\right)-A_{i}\left(t\right)\left[\alpha u_{0}\left(t\right)+\sum_{i\in N}\beta_{i}u_{i}\left(t\right)\right]\\ -\mu_{i}u_{i}^{2}\left(t\right)-\nu_{i}u_{0}^{}\left(t\right)\left(u-u_{i}^{}\left(t\right)\right)+\rho_{i}\tilde{x} \end{array}\right.,$$

Then, we obtain

(46) $$A_{i}\left(t\right)=\frac{e^{\left(r-\varepsilon \right)\left(T-t\right)}+\rho_{i}}{r-\varepsilon }.$$

Using Equation (44), we can derive the optimal resource strategies:

(47) $$u_{i}^{*}\left(t\right)=\frac{v_{i}u_{0}\left(t\right)-\beta_{i}A_{i}\left(t\right)}{2\mu_{i}},$$

where $A_{i}\left(t\right)$ is given by Equation (46).

Hence, Lemma 2. follows.
